# 200 years from the first documented outbreak: Dying of cholera in the Near East during 2022 (recent data analysis)

**DOI:** 10.7189/jogh.13.03004

**Published:** 2023-01-14

**Authors:** Saverio Bellizzi, Wiem Abdelbaki, Giuseppe Pichierri, Luca Cegolon, Christian Popescu

**Affiliations:** 1 University of Sassari, Sassari, Italy; 2 College of Engineering and Technology, American University of the Middle East, Kuwait; 3 Microbiology, Torbay and South Devon NHS Foundation Trust, Torquay, UK; 4 University of Trieste, Department of Medical, Surgical & Health Sciences, Trieste, Italy; 5 Occupational Medicine Unit, University Health Agency Giuliano-Isontina (ASUGI), Trieste, Italy; 6 University Medical Center Goettingen, Goettingen, Germany

The global pandemic spread of cholera from its ancestral home in Bengal was first documented in 1817, an event since recognized as the beginning of the first pandemic [1]. During the third pandemic, which ravaged London in 1854, John Snow conducted his pioneering epidemiologic studies and implemented one of the first evidence-based Water, Sanitation, and Hygiene (WASH) interventions [1].

After decades of decline, the fast-paced cholera outbreaks recently declared in Lebanon and Syria marked an unwelcome comeback in the Near East. Boosted by complex health emergencies, prolonged conflict, deteriorating economic conditions leading to poor water and sanitation systems, and climate change, cholera is now spreading at unprecedented levels in the region. Eight of 22 countries in the WHO Eastern Mediterranean Region have reported cases and deaths of cholera and acute watery diarrhea [2].

The Government of Syria reported the first case of cholera on the August 25, 2022. As of October 29, 2022, The Ministry of Health reported 30 219 suspected cases, including 85 deaths to date at a case fatality rate of 0.3% [3]. Moreover, Lebanon reported its first cholera outbreak in Lebanon since 1993 on October 5, 2022 [4], with 3042 suspected and confirmed cases, including 18 deaths by November 10, 2022 [5]. Iraq declared its outbreak on June 19, 2022, with cholera cases spiralling during summer with 865 confirmed cases [6].

While triggers for cholera outbreaks like poverty and conflict are long-existing issues, climate change and economic hardship due to COVID-19, high oil prices, and other factors represent a growing threat. Extreme climate events like floods, cyclones, and droughts are becoming more frequent, further reducing access to safe water, while economic hardship limits coping mechanisms for vulnerable populations. Water is a key driver of cholera, both during droughts and floods. During droughts, populations cannot access their usual water sources and must resort to other, at times unsafe ones [7]. Droughts are a constant threat in the Near East [8] and cause people to flee their homes; these migrations are associated with deteriorations of hygiene and sanitation, as well as additional risks like gender-based violence [9].

Cholera is deadly, but it is also preventable through vaccines and access to safe water and sanitation. More severe cases are also easily treatable with oral rehydration or antibiotics. Unfortunately, many people do not have access to these simple interventions. A striking example is provided by Syria, where more than 10 years of conflict have decimated civilian infrastructure and services, including health care facilities, water and sanitation systems, and electricity grids. The United Nations (UN) estimates that two-thirds of Syria’s water treatment plants, half of its pumping stations, and a third of its water towers have been damaged since 2011 [10].

Simultaneously, the International Coordinating Group (ICG) on vaccine provision is suspending the standard two-dose vaccination regimen in cholera outbreak response campaigns, resorting instead to a single-dose approach. The temporary use of a one-dose strategy due to a global shortage will allow more people to be vaccinated and provide them short- term protection [11]. While vaccines are a critical tool, they will not address the root causes for the increasing number and severity of outbreaks.

**Figure:**
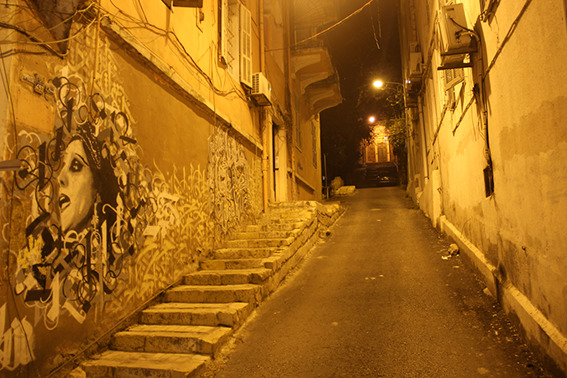
Photo: Streets of Beirut, Lebanon, at night. From Saverio Bellizzi’s own collection.

To sustainably end the increasing global transmission of cholera, the Global Task Force on Cholera Control (GTFCC), a partnership of more than 50 non-governmental organizations (NGOs), academic institutions, and UN agencies [12], launched the “The Global Roadmap to 2030” in 2017. The roadmap aims for a 90% reduction in cholera deaths and cholera elimination in 20 countries by 2030 [13]. Among the proposed interventions, WASH provides the long-term solution, while risk communication and community engagement (RCCE) is at the core of the strategy. Cholera can be prevented with access to safe water and sanitation. As clearly shown by COVID-19 and the Ebola experience in West Africa during 2014/2015, RCCE is probably the most powerful and cost-effective tool for protecting health care workers and populations. However, providing technical guidance to ensure proper clinical management practices, infection prevention and control, cholera testing protocols and surveillance systems remains critical. The total cost is estimated at US$11 annually per person at risk, with a return to investment ratio of 10 to 1 [14].

These past lessons are learned from painful experiences from contexts ranging from Haiti to Yemen [15-17]. National governments, alongside humanitarian and development actors heed these lessons, both to save lives and precious resources. Yet despite our knowledge on what works, case fatality rates during recent outbreaks in the Near East seem higher than usual. This is in part due to the sizes of the outbreaks: the larger the outbreak, the worse the outcomes. Climate events have also had an impact, as has the fact that outbreaks occurred in conflict-affected areas. Health resources have also been repurposed during the COVID-19 pandemic. These and other factors have played a role, while critical investments to fund the “The Global Roadmap to 2030” were not realized.

What would John Snow say, one and a half century after he implemented WASH interventions to stop cholera transmission in London, if he heard that people in 2022 were still dying of cholera? Quick and impactful action is needed.
